# Retrospective analysis of regional and metropolitan school food environments using Google Street View: A case study in New South Wales, Australia with youth consultation

**DOI:** 10.1002/hpja.930

**Published:** 2024-10-16

**Authors:** Kitty Tse, Michelle X. Zeng, Alice A. Gibson, Stephanie R. Partridge, Rebecca Raeside, Radhika Valanju, Emily McMahon, Bowen Ren, Fulin Yan, Margaret Allman‐Farinelli, Si Si Jia

**Affiliations:** ^1^ Sydney Nursing School, Faculty of Medicine and Health Discipline of Nutrition and Dietetics, The University of Sydney Sydney New South Wales Australia; ^2^ Faculty of Medicine and Health Menzies Centre for Policy and Economics, School of Public Health, The University of Sydney Sydney New South Wales Australia; ^3^ Charles Perkins Centre The University of Sydney Sydney New South Wales Australia; ^4^ Faculty of Medicine and Health, School of Health Sciences Engagement and Co‐Design Research Hub, The University of Sydney Sydney New South Wales Australia; ^5^ Faculty of Medicine and Health The Health Advisory Panel for Youth at The University of Sydney Sydney New South Wales Australia

**Keywords:** adolescents, food environment, Google Street View, obesity, schools, virtual audit

## Abstract

**Issue Addressed:**

Food environments surrounding schools have a strong influence on the adolescent's food choices. Moreover, the prevalence of diet‐related chronic diseases is higher in regional than metropolitan areas in Australia. Understanding school food environments in these different settings is crucial for informing future strategies to improve adolescent health.

**Methods:**

Google Street View was used to identify food outlets within 1.6 km around all secondary schools in Wagga Wagga and Blacktown in New South Wales which were selected as regional and metropolitan case study areas. Based on food outlet type, healthfulness categories were assigned, and Chi‐squared tests were performed. The Health Advisory Panel for Youth at the University of Sydney (HAPYUS) were engaged to obtain their perspectives on findings.

**Results:**

Unhealthful food outlets were consistently most prevalent around schools in Wagga Wagga and Blacktown over 17 years. In 2023, these were predominantly restaurants (19.4% vs. 21.1%), cafés (16.8% vs. 11.1%), fast‐food franchise outlets (15.1% vs. 17.4%) and independent takeaway stores (14.1% vs. 9.6%). No significant difference in healthfulness between regional and metropolitan areas was found. Youth advisors recognised price and social reasons as major contributors to food choices.

**Conclusions:**

Google Street View was used as a novel resource to examine school food environments in regional and metropolitan areas which have remained consistently unhealthful for nearly two decades.

**So What?:**

Unhealthful school food environments may encourage poor diets and exacerbate rates of adolescent overweight and obesity. Critical government action is needed to improve school food environments.

## 1 INTRODUCTION

Optimal nutrition during adolescence (10–19 years of age) is vital given that it is a life stage of significant physical and cognitive development and presents a critical opportunity to build a foundation for future good health.^1^ Despite this, currently 25% of Australian adolescents are living with overweight or obesity.^2^ This has been mainly attributed to the poor diet quality of adolescents: <10% are meeting the recommended daily consumption of vegetables and a significant proportion of their total daily energy intake is obtained from discretionary foods (i.e., energy‐dense, nutrient‐poor foods) and sugar‐sweetened beverages.^2^ Poor adolescent diet quality has been associated with increased risk of developing chronic conditions such as type 2 diabetes, cardiovascular diseases and kidney diseases.^3^, ^4^ Furthermore, a systematic review of 12 epidemiological studies revealed a significant positive association between poor diet and poor mental health in adolescents.^5^ Given the association between poor diet and poorer physical and mental health outcomes,^3^, ^5^ adolescent nutrition quality is therefore a significant public health issue especially if the establishment of suboptimal dietary and lifestyle habits is carried into adulthood.^6^

Adolescents residing in rural or regional areas have a higher prevalence of overweight compared with those living in metropolitan areas (28% and 23%, respectively)^2^ and generally at higher risk of developing chronic conditions compared with their metropolitan counterparts.^7^ This has been associated in part with the lower availability, accessibility, and affordability of nutritious food in regional and remote areas compared with metropolitan areas.^8^, ^9^ Previous research conducted in Australia also shows that the prevalence of mental health disorders in young people is higher in those living in regional and remote areas, compared with major cities.^5^, ^10^ The latest report published in 2022 from an inquiry into the health outcomes of regional, rural and remote New South Wales, unsurprisingly found greater disparities in health and health service access in these areas, compared with metropolitan counterparts.^11^

Food environments are the context in which physical, socio‐cultural, economic and political factors govern consumers' access and availability to food, influencing their food purchasing decisions, consumption habits and nutrition status.^12^ The food environment surrounding secondary schools is therefore considered a key influence on adolescent food choices and diet quality given their near‐daily exposure to this environment.^13^ Moreover, adolescence is a period where many children develop autonomy, self‐reliance and have increased purchasing power; hence, adolescents are particularly vulnerable to external influences from their food environment.^6^ As such, creating more healthful food environments around secondary schools is considered a key target area for current obesity strategies in Australia with particular focus for those living in regional areas.^14^, ^15^ Characterising food environments surrounding secondary schools, in regional and metropolitan settings, is essential to inform and advocate for healthful food environment policy.

Previous attempts to map Australian food environments have mostly been limited to urban settings and based on data extracted either from business directories, such as Yellow Pages and White Pages, or from ground‐truthing or physical street canvassing in a given area, often at a single time point.^16^, ^17^, ^18^ These methods are typically considered time‐consuming, susceptible to being outdated and resource‐intensive.^19^ Google Street View has been increasingly used in public health research examining the influence of built environments on health outcomes.^20^ Google Street View's ‘Time Machine’ feature allows for the viewing of time‐stamped historical imaging from 2007, to virtually audit how built environments have changed over time.^21^, ^22^ Google Street View can be utilised as an accessible source of retail outlet data for repeat cross‐sectional and longitudinal observations of changing food environments.^19^

As such, this study aimed to use Google Street View to examine changes in the food environment surrounding secondary schools in regional and metropolitan New South Wales, Australia from 2007 to 2023. A secondary aim was to investigate if there were any differences in the ‘healthfulness’ of food environments around schools in regional and metropolitan areas.

## 2 METHODS

### 2.1 Study design and methods

A longitudinal study was used to examine changes in the food environments around secondary schools in a regional (Wagga Wagga) compared with a metropolitan (Blacktown) local government area in NSW, Australia over 17 years from 2007 to 2023. We selected 2007 as the start date as this was the earliest time Google Street View was available.

### 2.2 Sample selection

To compare the food environments of a regional and metropolitan area, two regions of similar socioeconomic status (SES) but of different levels of remoteness (i.e., relative geographic access to services)^23^ were selected. The Socio‐Economic Indexes for Areas (SEIFA) was used to compare the SES among different local government areas. The city of Wagga Wagga, an ‘Inner Regional’ area,^23^ was selected as a representative of regional NSW with an Index of Relative Socio‐Economic Advantage and Disadvantage (IRSAD) score of 978.^24^ SEIFA files dating back to 2007 were unavailable. Therefore, the two areas were matched using the IRSAD scores in SEIFA files from 2011 and 2021. IRSAD scores were observed to remain stable over the study timeframe. The IRSAD score is used to rank areas from most disadvantaged to most advantaged and accounts for factors such as income, education and employment. The reference value for the whole of Australia is set to 1000 which indicates that scores below this value reflect a relative greater disadvantage.^25^, ^26^ Wagga Wagga's population estimate in 2023 was 68 716 compared with 59 493 in 2007, which reflects a 15.5% increase.^27^

The suburb of Blacktown, considered to be located within ‘Australia's Major Cities’,^23^ was subsequently selected as a representative for metropolitan NSW,^23^ sharing a similar IRSAD score of 993.^24^ In 2023, the population estimate for Blacktown City was 426 202, compared with 284 925 in 2007, which reflects a 49.6% increase.^28^

Eight secondary schools in Wagga Wagga and seven secondary schools in Blacktown were identified in 2023. Wagga Wagga schools were predominately co‐educational excepting one all‐girls' school; three were government‐funded (public) schools and five were independent (private) schools; five were secondary schools and three were combined schools (with primary and secondary students).^29^ In Blacktown there were four public, three private; four co‐educational; two all‐boys' schools and one all‐girls' school. Most schools were secondary schools except one combined school.^29^ All schools were present throughout the study period from 2007 to 2023.

### 2.3 Characterisation of local food environments around secondary schools

Our research catchment area was within 1 mile or 1.6 km around a school, generally considered a walkable distance for adolescents.^30^ Circleplot^31^ was used to generate a Keyhole Markup Language (KML) file that marked a 1.6 km radius around a school when uploaded into Google My Maps (Figure S1). This process was repeated for all identified schools.

Food outlet search terms were obtained from a list of over 4000 Google My Business Categories, publicly available online. Of this list, nine general search terms relevant to adolescent food choices^32^ were selected (‘Takeaway’, ‘Convenience Store’, ‘Bubble Tea’, ‘Café’, ‘Bakery’, ‘Shop’, ‘Restaurant’, ‘Grocery’ and ‘Market’) and used as input on Google My Maps. Any food outlets discovered within the 1.6 km radius were marked. This search strategy was further supplemented by manually checking the results from Google My Maps against results from Google Maps. In addition, a sample of 20% of food outlets was cross‐checked by two independent researchers to ensure all relevant food outlets were captured.

Food outlets located within hotels and social clubs, such as bottle shops and pubs were excluded as they were not considered as common places adolescents procured food. Retail history at all marked locations within the 1.6 km radius was visually examined using Google Street View's ‘Time Machine’ feature from 2007 to 2023. For food outlet records that could not be obtained from Google Street View, other methods were used including: Internet Archive Wayback Machine,^33^ to access archived versions of food outlet websites and/or online store directories, historical social media posts on Facebook and Instagram and dated local newspaper articles. Please see Figures S1–S3 for more details on this process.

### 2.4 Data collection

The following data on food outlets were collected from 2007 to 2023: ‘Address’, ‘Year’, ‘Food outlet name’ and ‘Food outlet category’ as listed in My Maps.

### 2.5 Food classification

Food outlets were further classified to be assessed for their potential impact on health using a version of a ‘Food Environment Score’ tool modified by Needham et al.^16^ and originally developed by Moayyed et al.^34^ in consultation with Australian public health and nutrition experts (Table S1). Food outlets were classified into one of 13 ‘Food Outlet Type’ categories in accordance to how closely an outlet adhered to a definition describing each outlet type. The Food Environment Score tool also assigns a health rating from −10 (least healthful) to 10 (most healthful) depending on the food outlet type and categorises food outlets into one of three broad ‘Health Categories’: ‘Healthful’ (H), ‘Less Healthful’ (LH) and ‘Unhealthful’ (UH), depending on their health rating, for example, ‘Starbucks’, described by Google Maps as a ‘coffee shop’, was classified as a ‘Café’ by the Food Environment Score tool, assigned a health rating of ‘0’ and classified as ‘Less Healthful’. Food outlets in the ‘less healthful’ category are typically independent restaurants and cafes which are run by small business owners and subject to wide variation in recipes for menu items.

### 2.6 Data analyses

Descriptive statistics were used to describe changes in the local food environments from 2007 to 2023 in Wagga Wagga and Blacktown by ‘Food Outlet Type’ (e.g., ‘Restaurant’, ‘Café’, ‘Bakery’ etc.) and by ‘Health Category’.

Chi‐squared tests of independence (*χ* ^2^ test) were performed in Microsoft Excel (Version 18.0) [Computer Software] to determine any significant differences in the health profile of food environments (i.e., proportion of healthful/less healthful/unhealthful food outlets) between Wagga Wagga and Blacktown in 2007 and between Wagga Wagga and Blacktown in 2023. Additional Chi‐squared tests were performed to assess whether the health profile of food environments had significantly changed between 2007 and 2023 in Wagga Wagga and between 2007 and 2023 in Blacktown. A significance level of *p* < .05 was used.

### 2.7 Adolescent consultation exercise

In August 2023, authors (RR, SJ, SRP) presented an overview of the study and its findings to an established youth advisory group of 16 adolescents aged 13–18 years at the time of joining in October 2021, residing in New South Wales, Australia (Health Advisory Panel for Youth at the University of Sydney [HAPYUS]).^35^ Full details about the youth advisory group are available elsewhere as part of a formal evaluation of the group, including remuneration and all skill development and training that were provided.^35^ This approach was supported by the NHMRC Statement on Consumer and Community Involvement in Health and Medical Research.^36^

Research that involves young people has been shown to have multiple benefits, including more insightful data interpretation by translating meaning to adult researchers and wider and more effective research dissemination.^37^ The youth advisory group has previously contributed their insights to multiple research projects focused on adolescent health^38^, ^39^, ^40^ and led a perspective article on their experiences working in chronic disease prevention research.^41^ This study was informed by their concerns about the rise of fast‐food chains and the implications for young people's health outcomes that were voiced in their published perspective article.^40^, ^42^, ^43^ As such, for this study, youth advisors were engaged after data collection and analyses to obtain their perspectives of the change in food environments surrounding high schools over time, providing rich insight on how the study findings may impact the health of adolescents and potential areas for action. Four members of the youth advisory group contributed to a 500‐word statement which is included in the results section.

### 2.8 Ethical considerations

Ethics approval was not required. This research involved the use of existing collections of publicly available data from Google Street View. Moreover, the youth advisory group members who contributed to the 500‐word statement are members of the research team and are included as co‐authors—similar to previously published research.^38^, ^39^, ^40^ When youth advisors participated in research in a role such as co‐researcher or consultation exercise, ethical approval was not required as none of their personal health data or demographic data were collected or reported as part of the study. Specific advice received from The University of Sydney Human Research Ethics Committee, who supported this approach.

## 3 RESULTS

### 3.1 Changes in food environments around secondary schools by food outlet type

**FIGURE 1:**
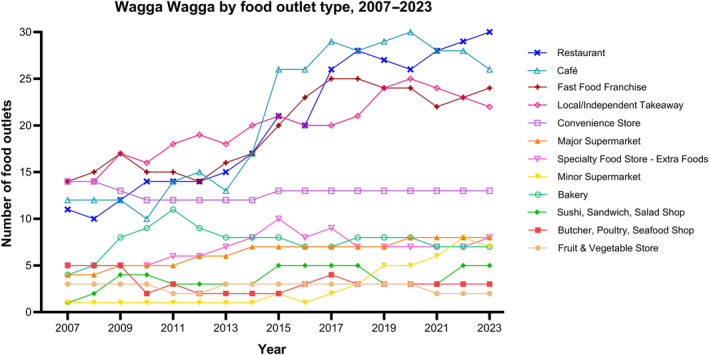
Changes in the number of food outlets, by food outlet type, within 1.6 km radius of all secondary schools identified in Wagga Wagga from 2007 to 2023.

From 2007 to 2023, the total number of identified food outlets located near secondary schools increased by 76% (88–155 outlets) in Wagga Wagga and 78% (152–272 outlets) in Blacktown (Table S1). As of 2023 in Wagga Wagga, the most prevalent food outlet types were restaurants (19.4%), cafes (16.8%), fast‐food franchises (15.1%) and independent takeaway outlets (14.1%) (Figure 1). These outlet types have consistently remained the most prevalent over a 17‐year period, although restaurants and cafes have since surpassed the number of fast‐food franchises and independent takeaway stores which previously dominated the food environment in 2007 (comprising over 30% of total food outlets combined). Most types of food outlets in Wagga Wagga increased between 2007 and 2023 (Table S1) with the largest increases being observed for minor supermarkets (+600%), sushi, salad or sandwich shops (+400%) and restaurants (+173%). Conversely, the total number of fruit and vegetable stores (−33%), butcher, poultry, seafood shops (−40%) and convenience stores (−7%) declined over this period.

**FIGURE 2:**
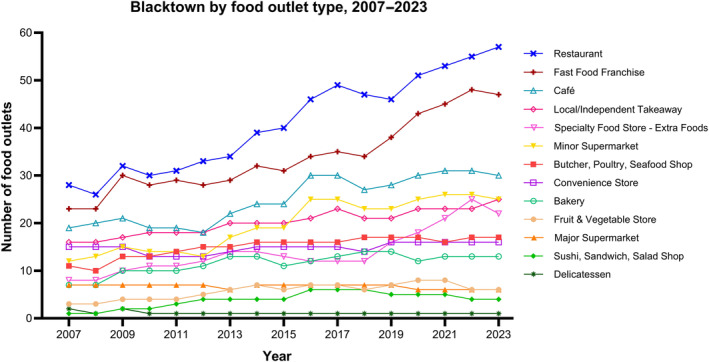
Changes in the number of food outlets, by food outlet type, within 1.6 km radius of all secondary schools identified in Blacktown from 2007 to 2023.

In Blacktown, restaurants were similarly observed to be the most common type of food outlet over 17 years (18.4% and 21.1% of total outlets in 2007 and 2023, respectively) followed by fast‐food franchises (15.1% and 17.4%), cafés (12.5% and 11.1%) and minor supermarkets (7.9% and 9.6%) (Figure 2). Nearly all types of food outlets increased in Blacktown between 2007 and 2023 (Table S1) with the largest increases being observed in sushi, salad or sandwich shops (+300%), specialty food stores (+175%), minor supermarkets (+117%), restaurants (+104%) and fast‐food franchises (+104%). Only delicatessens (−50%) and major supermarkets (−14%) declined over this period.

### 3.2 Health profile of food environments around secondary schools

**FIGURE 3:**
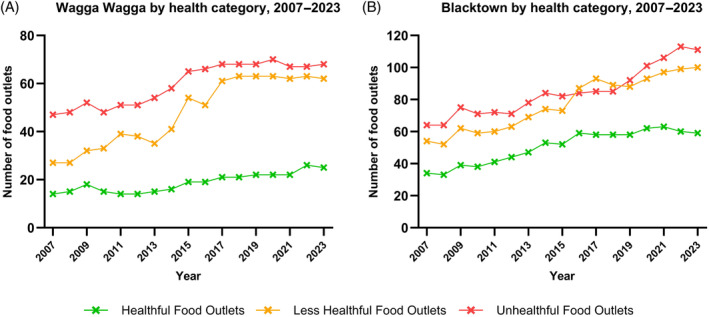
Comparison of the healthfulness of food outlets within 1.6 km radius of all secondary schools identified in (A) Wagga Wagga and (B) Blacktown from 2007 to 2023.

**FIGURE 4:**
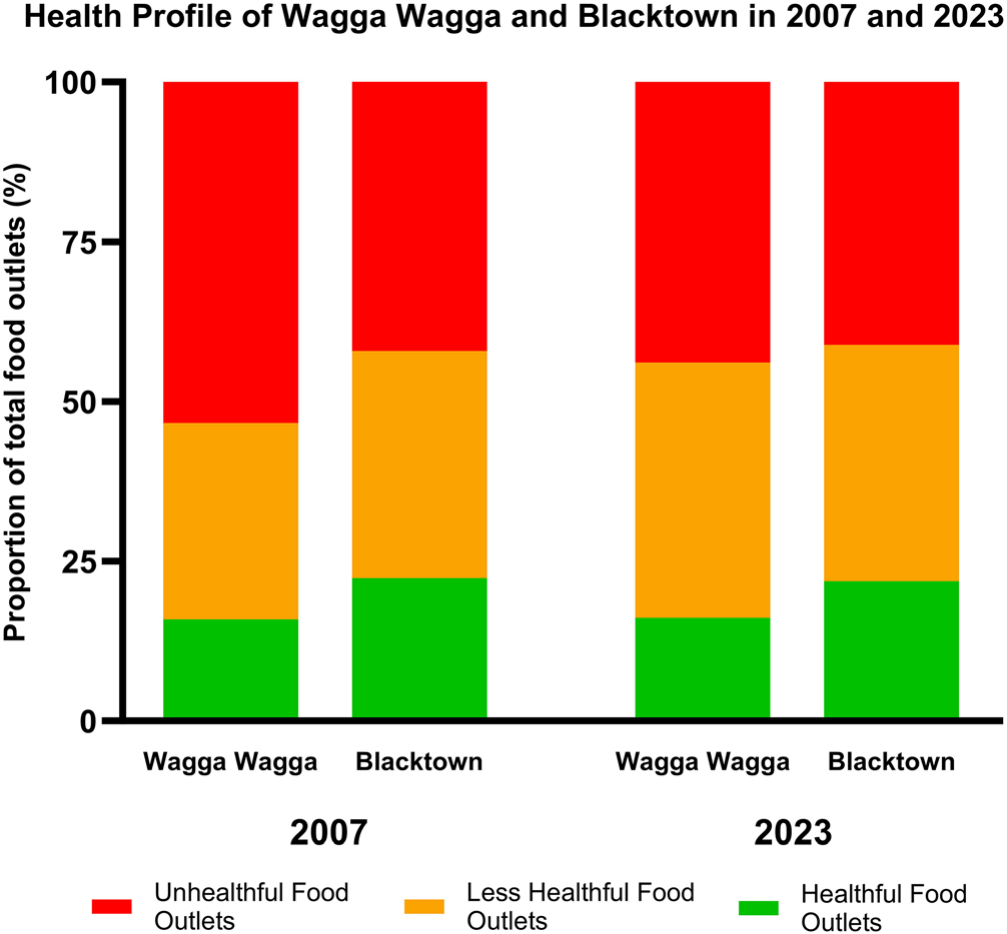
Health profile of food outlets within 1.6 km radius of all secondary schools identified in Wagga Wagga and Blacktown in 2007 and 2023.

From 2007 to 2023, the majority of food outlets within a 1.6 km radius of all identified secondary schools in Wagga Wagga were categorised as unhealthful (53% and 44% of total outlets in 2007 and 2023, respectively), followed by less healthful (31% and 40%) with healthful food outlets being the least common (16% and 16%) (Figures 3A and 4). Similar findings were reported in Blacktown from 2007 to 2023 with the highest proportion of food outlets categorised as unhealthful (42% and 41% in 2007 and 2023, respectively). As shown in Figure 3B, we note that there was a steep increase in unhealthful food outlets between 2018 and 2022 in Blacktown. This contrasts to Wagga Wagga's unhealthful outlets which appeared to plateau from around 2014 (Figure 3A). In 2007, 36% of food outlets and 37% of food outlets in 2023 were less healthful and the proportion of healthful food outlets remained stable at both time points—comprising 22% of food outlets by healthfulness category.

When comparing differences in the health profile of food environments around secondary schools in our study areas, no significant difference was reported between Wagga Wagga (*n =* 88) and Blacktown (*n =* 152) in 2007 (*X* ^2^ = 3.09, *p* = .21); or between Wagga Wagga (*n =* 155) and Blacktown (*n =* 270) in 2023 (*X* ^2^ = 2.03, *p* = .36) (Figure 4). When comparing changes in the health profile of food environments between the starting and endpoint of our study period, no significant change was reported between 2007 (*n =* 88) and 2023 (*n =* 152) in Wagga Wagga (*X* ^2^ = 2.41, *p* = .30); or between 2007 (*n =* 155) and 2023 (*n =* 270) in Blacktown (*X* ^2^ = 0.096, *p* = .95) (Figure 4).

### 3.3 Consultation exercise—Youth advisors' statement

Youth advisors produced a 500‐word statement as shown in the Box 1. Adolescents agreed that physical proximity to unhealthful food outlets around schools was a key contributor to unhealthful eating habits however, they have also recognised social and economic factors which play a significant role in shaping poor diets. The following quotes from their combined statement reflect social and economic concerns: (i) *‘[We] observed on a daily basis young people were opting to travel to the closest food court for fast food, or in some extreme cases, even order fast food via a meal delivery app, to be delivered to the school—as opposed to choosing the closest outlet’*; (ii) *‘Overpriced foods in schools with some drinks costing 2× more than a heavily discounted KFC meal, no meaningful difference between foods sold at canteens and those sold at fast food outlets—heavily processed, packaged snacks, pre‐made reheated foods’*; (iii) *‘Most unhealthful food outlets choose a location that is not only close to schools but also close to major shopping centres and food courts … ideal for an after‐school social catchup as it appears to accommodate the needs of a large group of people’*. Consultation findings revealed the importance of social and economic factors that must be analysed in addition to adolescents' physical proximity to food outlets around their schools.

BOX 1 **Youth advisors' combined statement on the study findings and their experiences of school food environments**

The results of this study indicate that fast food chains and unhealthful food outlets are a growing influence on the food environments, and consequently, eating habits, young people are exposed to. We agree with the findings that physical proximity to schools is one key correlation of this trend but based on our lived experiences with food environments around schools, we believe that the concentration of unhealthful outlets, and the mental attraction toward them, stemming from young people's social and economic concerns, also plays a significant role in encouraging unhealthful eating habits in young people. We thus call upon policy makers to pursue a long‐term solution that is addressing these key concerns of young people to promote healthful eating.

Physical proximity between schools and fast food chains, although important, is not the determining factor in young people's eating choices. It is clear that no food outlet could be physically closer to students than the school canteens, yet we observed on daily basis young people opting to travel to the closest food court for fast food, or in some extreme cases, even order fast food via food delivery platforms to be delivered to the school, as opposed to choosing the closest outlet. This highlights prominent issues in school systems which is firstly, the overpriced nature of foods sold in schools, with some drinks costing almost as much as a heavily discounted KFC meal. And secondly, there lacks any meaningful difference between food sold at canteens and those sold at fast food outlets, with most foods sold at canteens being heavily processed, packaged snacks, or pre‐made reheated foods. Given the economic constraints young people face, it is only logical that fast foods become the more popular choice, and recognising the demand, it is inevitable that unhealthful food outlets would want to capture this large market unable to be satisfied by the school system.

Capitalising on this opportunity, most unhealthful food outlets choose a location that is not only close to schools, but also close to other such outlets, preferencing major shopping centres and food courts. The high concentration of fast food provides young people with the illusion of choice, making food courts ideal for an after school social catchup as it appears to accommodate the needs of a large group of people. The social pressure, in addition to economic pressures and the unfortunate truth that most young people have parents still at work after school and do not know how to cook healthful food themselves, we believe ultimately led to the concerning trend highlighted in this study.

Young people's eating habits are clearly affected by social, economic and family pressures and fast food chains are evidently utilising these pressures to make profits while governments attempt to blame unhealthful habits purely on lack of education. To promote sustainable healthful lifestyles, governments should take immediate action in enacting policies that ensures adequate funding for schools and healthful and cheap foods are sold at canteens.

## 4 DISCUSSION

This study used Google Street View to retrospectively analyse and compare changes in the food environment around secondary schools in Wagga Wagga and Blacktown from 2007 to 2023. Overall, it was found that the total number of food outlets increased greatly in the food environments surrounding secondary schools of Wagga Wagga and Blacktown over this 17‐year period with similar increases (~76%–78%) reported in both regions. The most common food outlet types over the study period were all classified as either less healthful or unhealthful such as restaurants, cafés, fast‐food franchise outlets and local takeaway stores. No significant difference was found when comparing the healthfulness of food environments between our regional and metropolitan case study areas in 2007 and 2023 despite varying levels of remoteness. Additionally, no significant differences were found when comparing the health profiles of food environments between 2007 and 2023 in both regional and metropolitan case study areas. The consultation exercise with youth revealed that price and social reasons were key influences on unhealthful food purchases before, during and after school.

The expansion in the food environment near schools is likely driven by overall population growth in both Wagga Wagga and Blacktown and a response to increased demand for out‐of‐home foods. This study observed a 76% increase in total food outlets in Wagga Wagga and 78% increase in Blacktown. Since 2012, Wagga Wagga and Blacktown have both experienced population growths of 10% and 29%, respectively.^44^, ^45^ A similar association between population and food retail growth was observed in Australia by Needham et al.^16^ who reported total number of food outlets in Greater Melbourne increased by 35% from 2008 to 2016, outstripping the population growth of 21%. Our observed growth in number of food outlets in both study areas may also be a response to increased consumption and demand for takeaway foods. According to the 2015–2016 Australian Household Expenditure Survey, Australians on average spent 32% of their household food budget on eating out.^46^ In addition, market research reported in 2020 more than 75% of Australians ate takeaway food in a given 4‐week period, an increase from 64% the previous year.^47^ Given these trends, it is critical to monitor and explore how and if, food environments can determine food consumption and vice versa.

The types of food outlets available in the food environment play a crucial role in influencing an individual's food choices and nutrition status, particularly among adolescents as they become more autonomous and self‐reliant.^6^ Results from this study showed that restaurants, cafes, local takeaways and fast‐food franchises—food outlets categorised as less healthful and unhealthful, have consistently remained the most common types of food outlets surrounding secondary schools in Wagga Wagga and Blacktown over time. At the same time, healthful food outlet types such as sushi, sandwich and salad shops have observed large increases in both areas, although they constitute a very small proportion of their respective food environments. This high proportion of unhealthful food outlets to healthful food outlets has been similarly demonstrated in a previous study that assessed both internal and external food environment of primary and secondary schools in three low SES regional local government areas in Tasmania, Australia.^48^ The results showed an abundance of unhealthful food outlets surrounding the schools in all three local government areas with high ratios of unhealthful‐to‐healthful food outlets of 16:1, 6:1, and 9:1, respectively.^48^ Overseas studies from New Zealand and Canada also demonstrated similar results that a high density of unhealthful food outlets was found within walking proximity to schools, especially in low SES areas.^49^, ^50^ Studies have established a link between adolescents purchasing food from external food retailers around schools with intake of energy‐dense, nutrient‐poor foods.^51^, ^52^ As such obesogenic food environments around secondary schools in both Wagga Wagga and Blacktown are likely to promote unhealthful food choices and dietary patterns among students that may contribute to the current prevalence of overweight and obesity and poor mental health in Australian adolescents.

No significant differences in the health profiles of food environments were found between our regional and metropolitan study areas. These findings align with similar studies that have investigated the impact of urban‐regional locality on food environments. In a study conducted by Thornton and colleagues in Victoria, Australia, no significant differences in the availability of fast food restaurants were observed across varying levels of urbanisation.^53^ Likewise, research from the Netherlands showed that most food outlet types had no significant differences between highest and lowest urbanisation level neighbourhoods, except for Convenience Stores and Supermarkets.^54^ We theorised that the similar SES levels between our study areas played a larger role than urbanisation, in shaping the profiles of our Wagga Wagga and Blacktown food environments. Smets and Vandevijvere^55^ have found that food environments near schools from areas with lower SES were generally more unhealthful regardless of urbanisation levels. Thus, further studies investigating the regional‐urbanisation variance in food environments must also consider the impact of SES in shaping the health profile of such environments.

Moreover, the consultation exercise conducted with youth advisors in this study revealed that the relative affordability of fast‐food outlets and ideal setting of these outlets as socialising spots after school, were key influences on young people's food choices. These insights are widely supported by the literature. A qualitative study from Queensland, Australia revealed high availability, affordability, and peer pressure were among factors that impacted adolescents' unhealthful dietary behaviours around their secondary school environments.^56^ Similarly, a qualitative study from Ireland found that cost, convenience and choice were important factors to adolescents' preferences for food outlets and foods, with more healthful options perceived as higher cost and more deals were offered for high‐fat and high‐sugar foods.^57^ In addition, a thematic synthesis of 26 articles showed that young people use food to foster their social connections and relationships.^58^ These are important considerations that should be accounted for in future research and policies to improve the healthfulness of secondary school food environments.

### 4.1 Strengths and limitations

Limitations of our research were mainly related to the constraints of Google Street View, particularly with image availability and quality. In Australia, there was a particularly large data gap in historical Google Street View imagery between 2010 and 2014 in both study areas. Moreover, imagery was mostly unavailable for food outlets in multi‐level building structures such as shopping centres, business parks or for certain street layouts inaccessible by road by Google Street View data collectors. Image quality could also be hampered by obstructions, such as large trucks and foliage, and poor image resolution (pre‐2009) that limited the visibility of storefronts required for outlet identification. In cases where food outlet data was unavailable from Google Street View, we resorted to other methods including food outlet websites and/or online store directories archived via the Wayback Machine, historical social media posts and dated local newspaper articles.

Furthermore, we acknowledge that there are different buffers commonly used to explore food environments such as 800 m or 1 km which we did not explore. A wider radius of 1.6 km may have inflated the number of food outlets which high school students were exposed to. Despite this, the consultation exercise conducted with our youth advisory group revealed that adolescents would travel a considerable distance to food places such as a food court in a nearby shopping centre. Moreover, Australian health geographers have used 1.6 km catchments in studies to reflect a walking distance of 15–20 min which indicates that this was an appropriate measure to use.^59^ We also note that due to limited resources, this study has only used Euclidean distances to measure school food environments. We recognise that road or street network buffers are a another commonly used approach to indicate exposure to food outlets. Despite this, multiple studies in the literature have exclusively used Euclidean buffers^60^, ^61^, ^62^ and research also shows that these two measures are largely comparable.^63^

In addition, there were also some concerns regarding the Food Environment Score tool in accurately assessing the healthfulness of food outlets as it does not account for the nutritional quality of menu items of food outlets. This issue is compounded by the known food preferences of adolescents toward discretionary foods^64^; adolescents may be more inclined to purchase discretionary foods in food outlets including those classified as healthful (e.g., supermarkets). As such, there may be an underestimation regarding the negative health potential of the adolescent food environment in our study areas.

Despite these limitations, Google Street View and the Food Environment Score tool were still the most time‐ and cost‐efficient methods to achieve the research aims of this study. Compared with previous food environment studies which purely relied on data sourced from business directories, the usage of Google Street View as our primary data source (i.e., virtual ground–truthing methodology), in addition to our secondary data sources (i.e., digital business directories, social media, dated newspaper articles) improved the comprehensiveness and accuracy of the data in this study.^19^ Furthermore, conducting a repeat cross‐sectional study that examined food environment changes over 17 years provided a more comprehensive snapshot of food outlet trends and changes in Australia. In addition, not many Australian studies to date have examined the food environment surrounding secondary schools. This study could provide insights into the current food environment around secondary schools, in both regional and metropolitan settings, and its changes over the past 17 years for state and federal governments to consider when planning strategies to address adolescent health and nutrition.

## 5 CONCLUSION

Food outlets surrounding schools are an important element of the adolescent food environment and a key influence on their food choice, diet quality and nutrition status. This study identified that the total number of food outlets has increased in the food environments surrounding secondary schools in a regional and metropolitan area case study over time. Additionally, unhealthful and less healthful food outlets have consistently constituted a much larger proportion of the food environments near secondary schools in our regional and metropolitan study areas. These findings uphold the need to support the provision of healthful food outlets around schools, in regional and metropolitan settings and should inform Australian preventive health and nutrition strategies targeting adolescents.

## AUTHOR CONTRIBUTIONS

**Kitty Tse:** Data collection; data analysis and interpretation; writing—original draft preparation. **Michelle X. Zeng:** Data collection; data analysis and interpretation; writing—original draft preparation. **Alice A. Gibson:** Conceptualisation; supervision; writing—review and editing. **Stephanie R. Partridge:** Conceptualisation; supervision; writing—review and editing. **Rebecca Raeside:** Supervision; writing—review and editing. **Radhika Valanju:** Investigation; data interpretation. **Emily McMahon:** Investigation; data interpretation. **Bowen Ren:** Investigation; data interpretation. **Fulin Yan:** Investigation; data interpretation. **Margaret Allman‐Farinelli:** Supervision; writing—review and editing. **Si Si Jia:** Conceptualisation; supervision; writing—review and editing.

## FUNDING INFORMATION

S.J. is supported on a Australian Government's Research Training Program Stipend Scholarship and a King and Amy O'Malley Trust Foundation. The funding is a top‐up postgraduate research scholarship. S.R.P. is supported by a the National Heart Foundation. The funding is a Future Leader Fellowship [Grant number: 106646]. A.A.G. is supported by Investigator Grant from the Australian National Health and Medical Research Council (NHMRC) (AAP1173784). This research did not receive any specific grant from funding agencies in the public, commercial, or not‐for‐profit sectors.

## CONFLICT OF INTEREST STATEMENT

The authors declare no conflicts of interest.

## Supporting information


**Data S1.** Supporting Information.

## Data Availability

The data that support the findings of this study are available from the corresponding author upon reasonable request.
