# Supplementary material for: Retrospective analysis of regional and metropolitan school food environments using Google Street View: A case study in New South Wales, Australia with youth consultation
Source: Health Promot J Austr. 2024 Oct 16;36(2):e930. doi: 10.1002/hpja.930 (PMC11806403; doi:10.1002/hpja.930)
Supplement: Supplementary file 1 — Data S1. Supporting Information. [file HPJA-36-0-s001.docx]

Supplementary Material

| 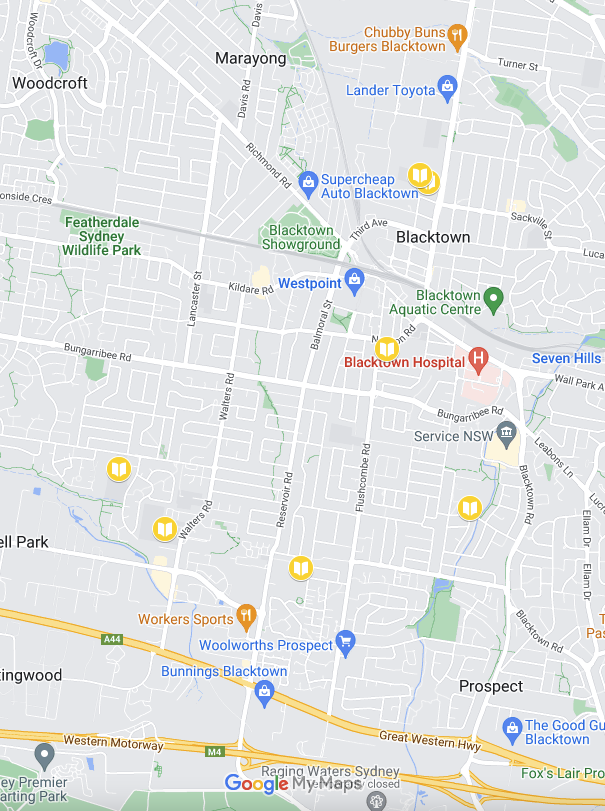**Figure S1a.** The secondary schools were first located on Google My Map | 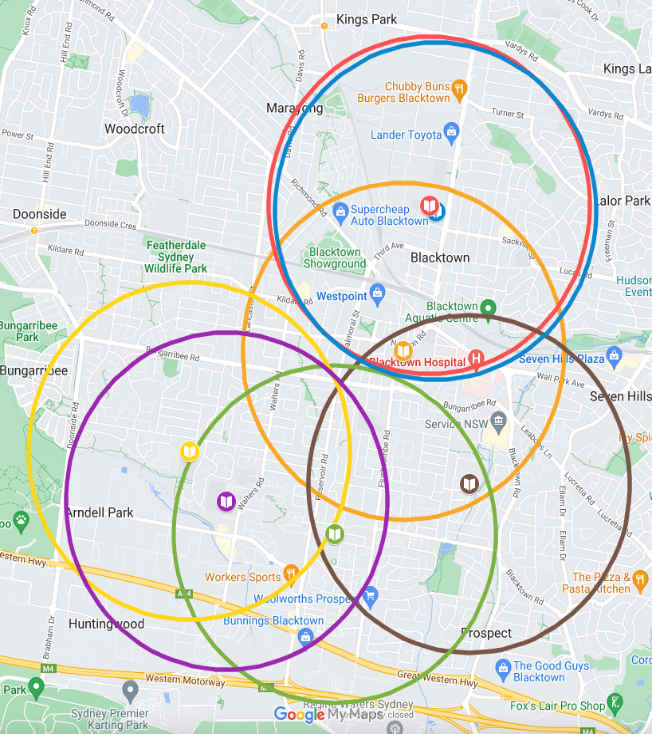**Figure S1b.** 1.6 radii were then placed around each secondary school. |
| --- | --- |
| 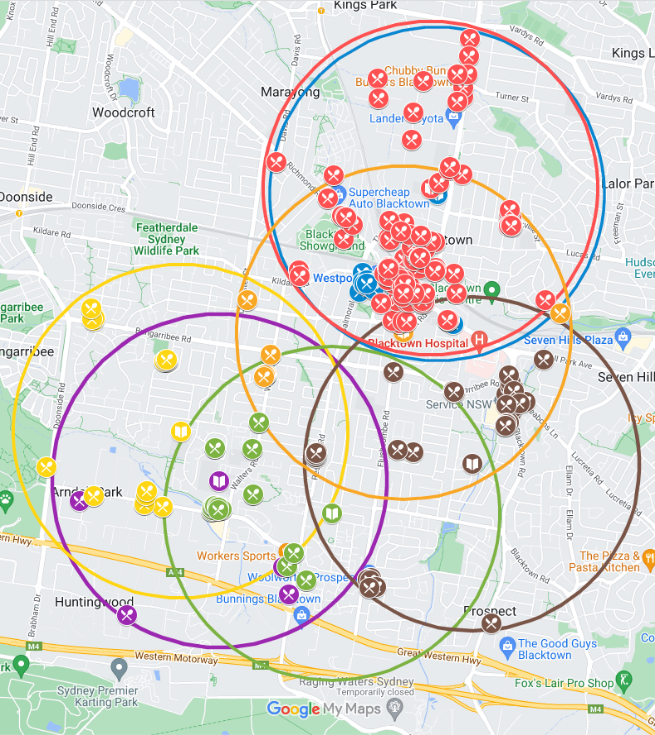**Figure S1c.** Food outlets that fell within the raii were pinpointed on the map for further investigation via Google Street View. |  |

**Figure S1.** Screenshot of sample research catchment area in Blacktown consisting of 1.6 radii surrounding schools generated in Google My Maps.


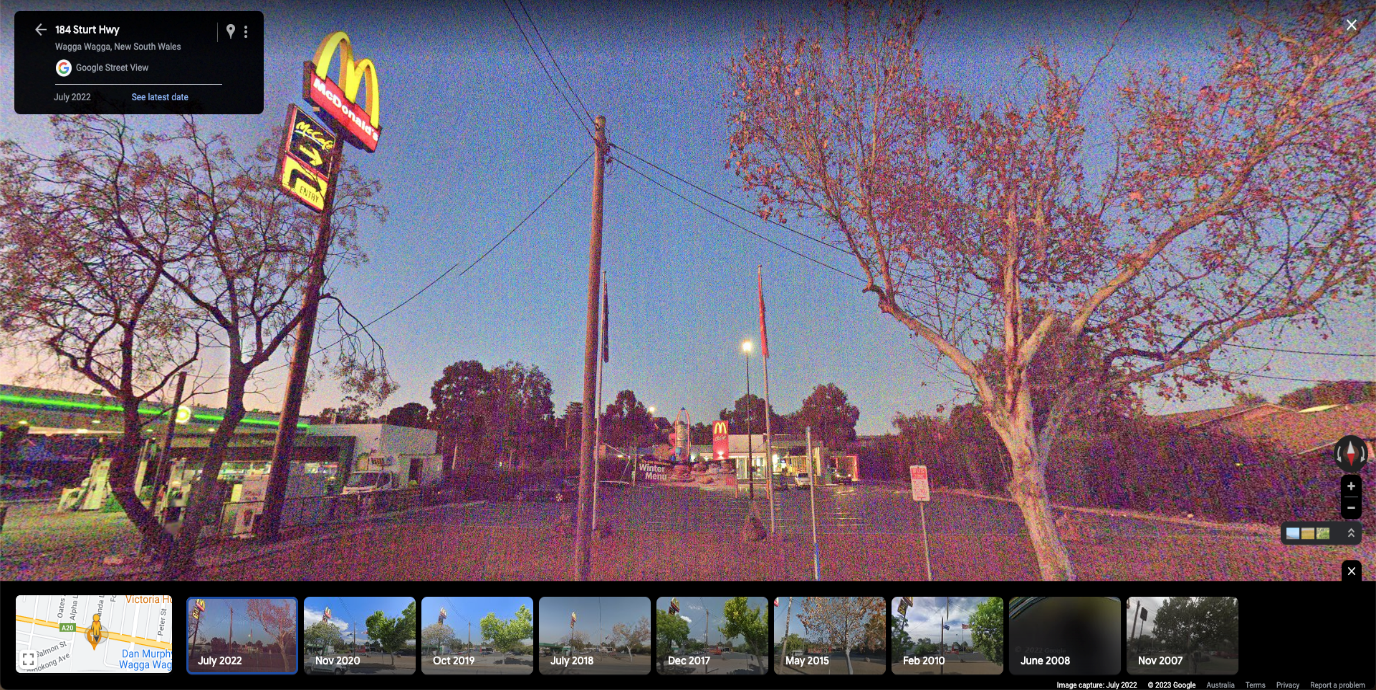
**Figure S2.** Screenshot exhibiting example of Google Street View and Time Machine for viewing historical food outlet data retrospectively from 2023 or the most recent street view image to 2007.


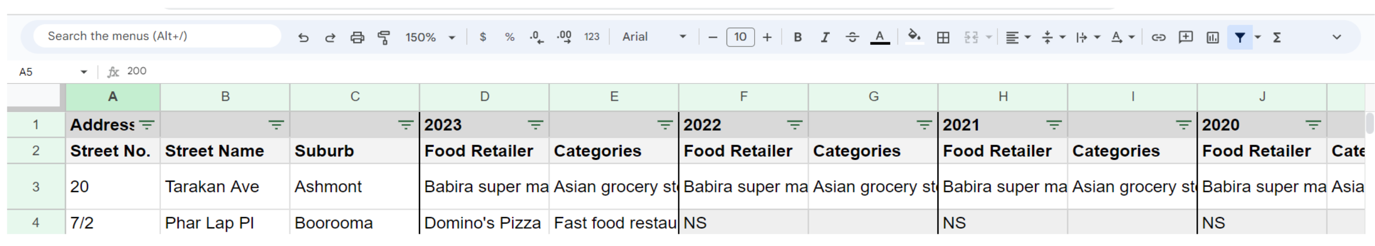
**Figure S3.** Screenshot exhibiting usage of Google Sheets to collect food outlet data (i.e., food outlet’s address, food retailer names, food outlet’s category) for food environments around secondary schools in Wagga Wagga and Blacktown over 17 years of time retrospectively from 2023 to 2007

Supplementary **Table 1:** Number and % change in the type of food outlets within 1.6 km radius of all secondary schools identified in Wagga Wagga and Blacktown from 2007-2023.

| **Food Outlet Type** | **2007** | **2008** | **2009** | **2010** | **2011** | **2012** | **2013** | **2014** | **2015** | **2016** | **2017** | **2018** | **2019** | **2020** | **2021** | **2022** | **2023** | **% Change^a^** |  |
| --- | --- | --- | --- | --- | --- | --- | --- | --- | --- | --- | --- | --- | --- | --- | --- | --- | --- | --- | --- |
| **Wagga Wagga** |  |  |  |  |  |  |  |  |  |  |  |  |  |  |  |  |  |  | |
| Total | 88 | 90 | 102 | 96 | 104 | 103 | 104 | 115 | 138 | 136 | 150 | 151 | 153 | 155 | 151 | 156 | 155 | +76% |  |
| Fruit & Vegetable Store | 3 | 3 | 3 | 3 | 2 | 2 | 3 | 3 | 3 | 3 | 3 | 3 | 3 | 3 | 2 | 2 | 2 | -33% |  |
| Butcher, Poultry,  Seafood Shop | 5 | 5 | 5 | 2 | 3 | 2 | 2 | 2 | 2 | 3 | 4 | 3 | 3 | 3 | 3 | 3 | 3 | -40% |  |
| Major Supermarket | 4 | 4 | 5 | 5 | 5 | 6 | 6 | 7 | 7 | 7 | 7 | 7 | 7 | 8 | 8 | 8 | 8 | +100% |  |
| Minor Supermarket | 1 | 1 | 1 | 1 | 1 | 1 | 1 | 1 | 2 | 1 | 2 | 3 | 5 | 5 | 6 | 8 | 7 | +600% |  |
| Sushi, Sandwich, Salad Shop | 1 | 2 | 4 | 4 | 3 | 3 | 3 | 3 | 5 | 5 | 5 | 5 | 3 | 3 | 3 | 5 | 5 | +400% |  |
| Bakery | 4 | 5 | 8 | 9 | 11 | 9 | 8 | 8 | 8 | 7 | 7 | 8 | 8 | 8 | 7 | 7 | 7 | +75% |  |
| Delicatessen | 0 | 0 | 0 | 0 | 0 | 0 | 0 | 0 | 0 | 0 | 0 | 0 | 0 | 0 | 0 | 0 | 0 | N/A |  |
| Café | 12 | 12 | 12 | 10 | 14 | 15 | 13 | 17 | 26 | 26 | 29 | 28 | 29 | 30 | 28 | 28 | 26 | +117% |  |
| Restaurant | 11 | 10 | 12 | 14 | 14 | 14 | 15 | 17 | 21 | 20 | 26 | 28 | 27 | 26 | 28 | 29 | 30 | +173% |  |
| Convenience Store | 14 | 14 | 13 | 12 | 12 | 12 | 12 | 12 | 13 | 13 | 13 | 13 | 13 | 13 | 13 | 13 | 13 | -7% |  |
| Specialty Store – Extra Foods | 5 | 5 | 5 | 5 | 6 | 6 | 7 | 8 | 10 | 8 | 9 | 7 | 7 | 7 | 7 | 7 | 8 | +60% |  |
| Independent Takeaway | 14 | 14 | 17 | 16 | 18 | 19 | 18 | 20 | 21 | 20 | 20 | 21 | 24 | 25 | 24 | 23 | 22 | +57% |  |
| Fast Food Franchise | 14 | 15 | 17 | 15 | 15 | 14 | 16 | 17 | 20 | 23 | 25 | 25 | 24 | 24 | 22 | 23 | 24 | +71% |  |
| **Blacktown** |  |  |  |  |  |  |  |  |  |  |  |  |  |  |  |  |  |  |  |
| Total | 152 | 149 | 176 | 168 | 173 | 178 | 194 | 211 | 207 | 230 | 236 | 228 | 238 | 256 | 266 | 272 | 270 | +78% |  |
| Fruit & Vegetable Store | 3 | 3 | 4 | 4 | 4 | 5 | 6 | 7 | 6 | 7 | 7 | 6 | 7 | 8 | 8 | 6 | 6 | +100% |  |
| Butcher, Poultry,   Seafood Shop | 11 | 10 | 13 | 13 | 14 | 15 | 15 | 16 | 16 | 16 | 16 | 17 | 17 | 17 | 16 | 17 | 17 | +55% | |
| Major Supermarket | 7 | 7 | 7 | 7 | 7 | 7 | 6 | 7 | 7 | 7 | 7 | 7 | 7 | 6 | 6 | 6 | 6 | -14% | |
| Minor Supermarket | 12 | 12 | 13 | 12 | 13 | 13 | 16 | 19 | 19 | 23 | 22 | 22 | 22 | 26 | 28 | 27 | 26 | +117% | |
| Sushi, Sandwich, Salad Shop | 1 | 1 | 2 | 2 | 3 | 4 | 4 | 4 | 4 | 6 | 6 | 6 | 5 | 5 | 5 | 4 | 4 | +300% | |
| Bakery | 7 | 7 | 10 | 10 | 10 | 11 | 13 | 13 | 11 | 12 | 13 | 14 | 14 | 12 | 13 | 13 | 13 | +86% | |
| Delicatessen | 2 | 1 | 2 | 1 | 1 | 1 | 1 | 1 | 1 | 1 | 1 | 1 | 1 | 1 | 1 | 1 | 1 | -50% | |
| Café | 19 | 20 | 21 | 19 | 19 | 18 | 22 | 24 | 24 | 30 | 30 | 27 | 28 | 30 | 31 | 31 | 30 | +58% | |
| Restaurant | 28 | 26 | 32 | 30 | 31 | 33 | 34 | 39 | 40 | 46 | 49 | 47 | 46 | 51 | 53 | 55 | 57 | +104% | |
| Convenience Store | 15 | 15 | 15 | 13 | 13 | 13 | 14 | 15 | 15 | 15 | 15 | 14 | 16 | 16 | 16 | 16 | 16 | +7% | |
| Specialty Store – Extra Foods | 8 | 8 | 10 | 11 | 11 | 12 | 14 | 14 | 13 | 12 | 12 | 12 | 16 | 18 | 21 | 25 | 22 | +175% | |
| Independent Takeaway | 16 | 16 | 17 | 18 | 18 | 18 | 20 | 20 | 20 | 21 | 23 | 21 | 21 | 23 | 23 | 23 | 25 | +56% | |
| Fast Food Franchise | 23 | 23 | 30 | 28 | 29 | 28 | 29 | 32 | 31 | 34 | 35 | 34 | 38 | 43 | 45 | 48 | 47 | +104% | |

^a^ % Change from 2007 to 2023
